# Supplementary material for: Multiple F-Box Proteins Collectively Regulate Cell Development and Pathogenesis in the Human Pathogen Cryptococcus neoformans
Source: J Fungi (Basel). 2022 Nov 29;8(12):1259. doi: 10.3390/jof8121259 (PMC9781138; doi:10.3390/jof8121259)
Supplement: Supplementary file 1 [file jof-08-01259-s001.zip › Supplementary figures.pdf]

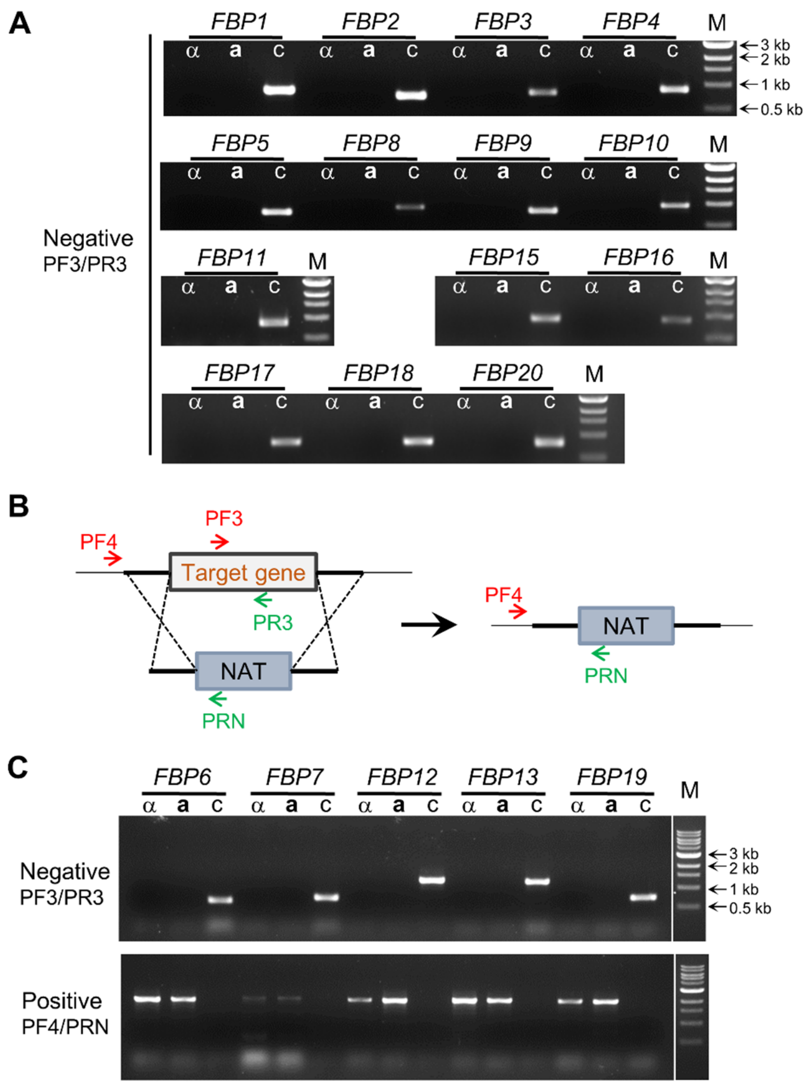

Figure S1. Deletion of *FBP6* (CNAG_03157), *FBP7* (CNAG_03421), *FBP12* (CNAG_05450), *FBP13* (CNAG_05454), and *FBP19* (CNAG_07551) in *C. neoformans*. (A) Colony PCR to confirm the mutants from the *Cryptococcus* deletion library. (B) Strategy for gene knockout by fusion PCR assays. Allele was replaced with the nourseothricin resistance marker *NAT1*. (C) Colony PCR screening and confirmation of the positive transformants. Primers used in this study are listed in Table S2.

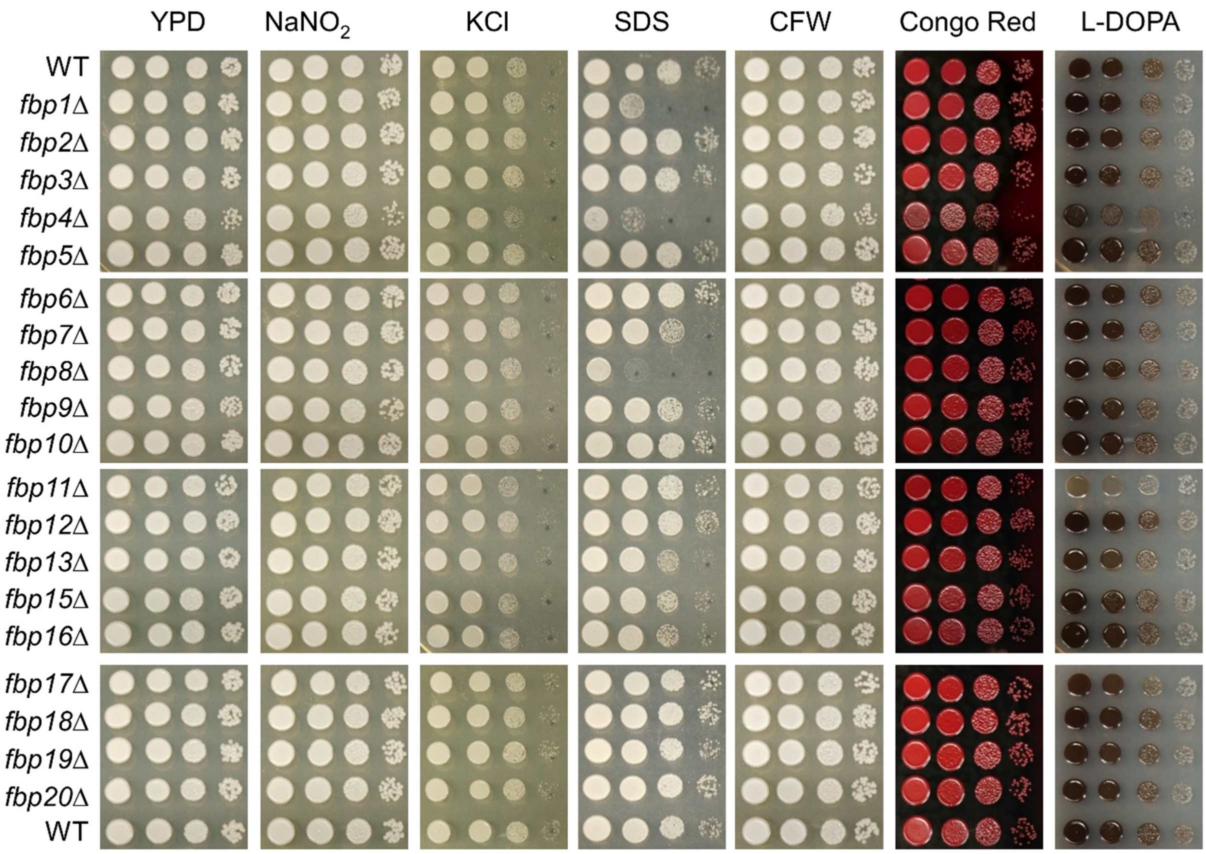

Figure S2. *In vitro* stress responses of F-box proteins in *C. neoformans*. Phenotypic assays were examined under different conditions as indicated. Overnight cultures were collected and resuspended to an optical density at 600 nm (OD600) of 10.0. Tenfold serial dilutions were made in ddH_2O, and 3 μl of each was plated. The plates were incubated at 37°C for 3 days before being photographed.

A 30 ℃

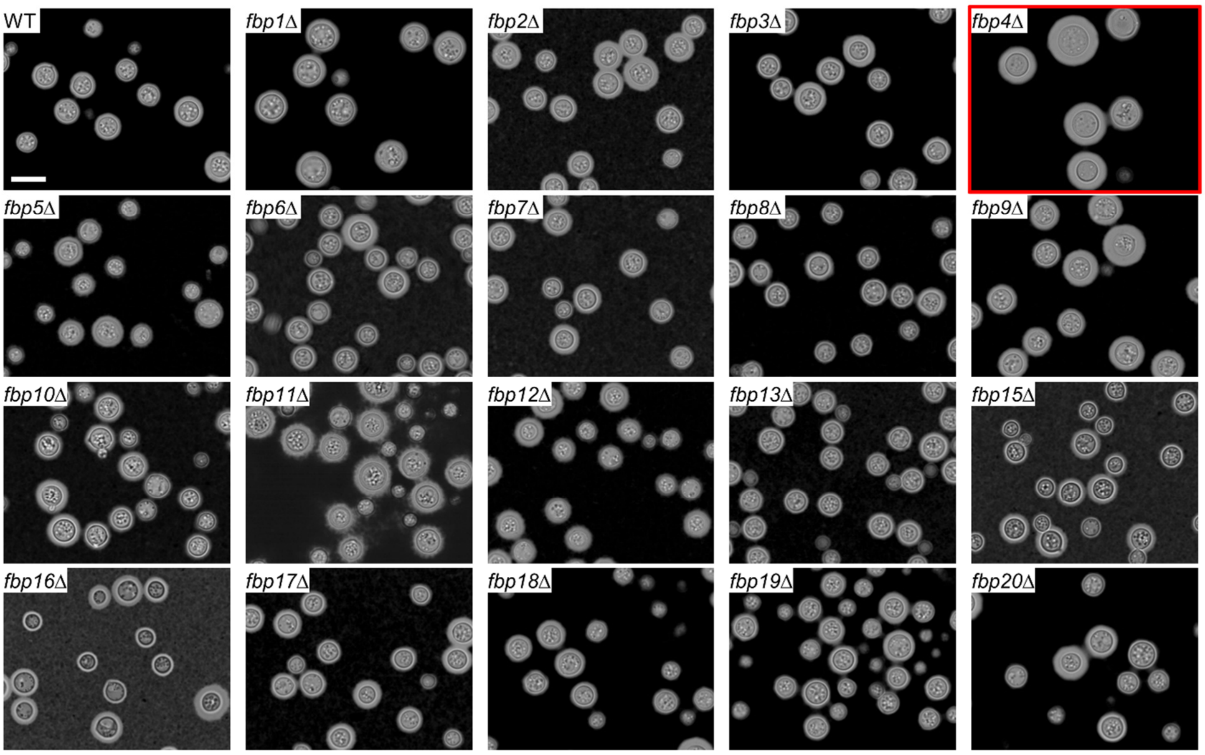
WT
fbp1Δ
fbp2Δ
fbp3Δ
fbp4Δ
fbp5Δ
fbp6Δ
fbp7Δ
fbp8Δ
fbp9Δ
fbp10Δ
fbp11Δ
fbp12Δ
fbp13Δ
fbp15Δ
fbp16Δ
fbp17Δ
fbp18Δ
fbp19Δ
fbp20Δ

B 37 ℃

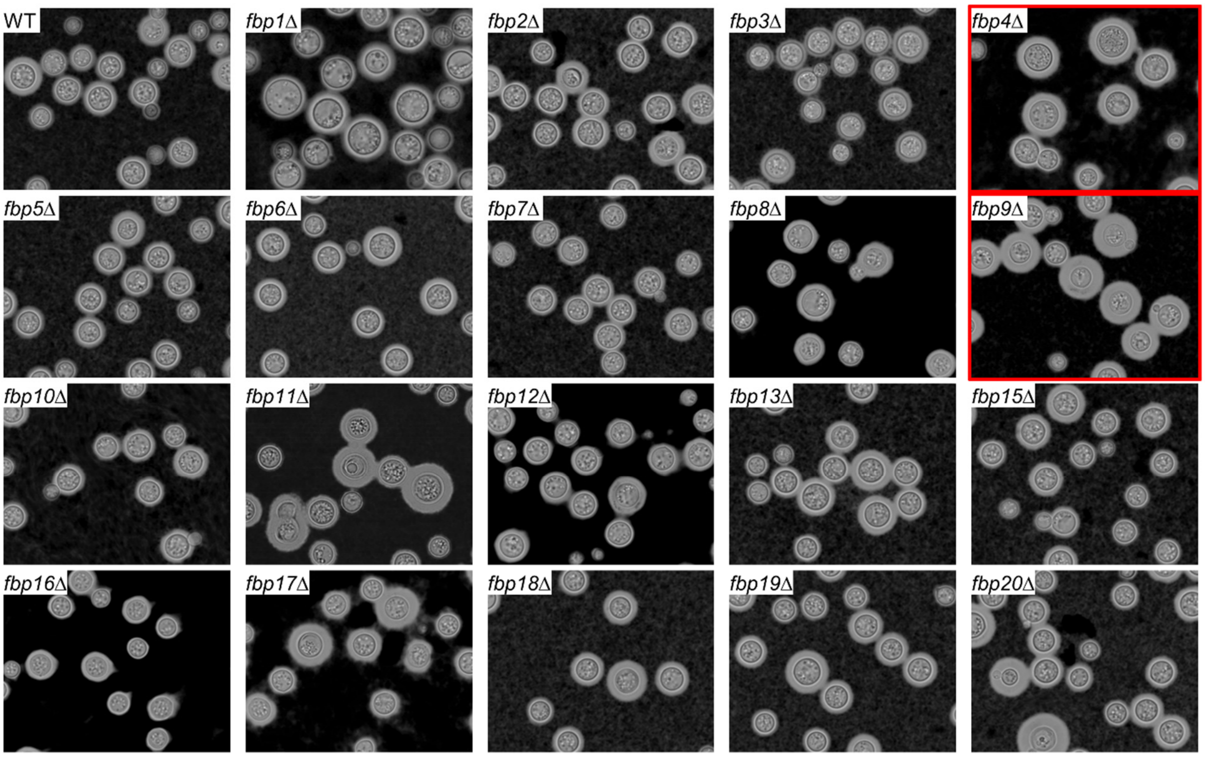
WT
fbp1Δ
fbp2Δ
fbp3Δ
fbp4Δ
fbp5Δ
fbp6Δ
fbp7Δ
fbp8Δ
fbp9Δ
fbp10Δ
fbp11Δ
fbp12Δ
fbp13Δ
fbp15Δ
fbp16Δ
fbp17Δ
fbp18Δ
fbp19Δ
fbp20Δ

Figure S3. Representative images to show capsule formation at 30°C (A) or 37°C (B) on DME medium. Capsule production was visualized by India ink staining after cells were grown on DME medium for 7 days.

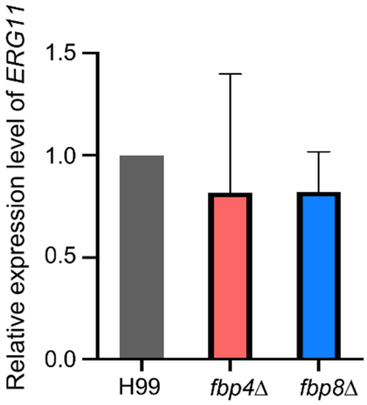

Figure S4. *ERG11* expression in F box protein deletion mutants and complimented strains analyzed by qRT-PCR. Samples were grown in YPD with 1 µg/mL Fluconazole for 4 hours at 30°C. Differential gene expression relative to GAPDH was calculated by the ΔΔCT method.

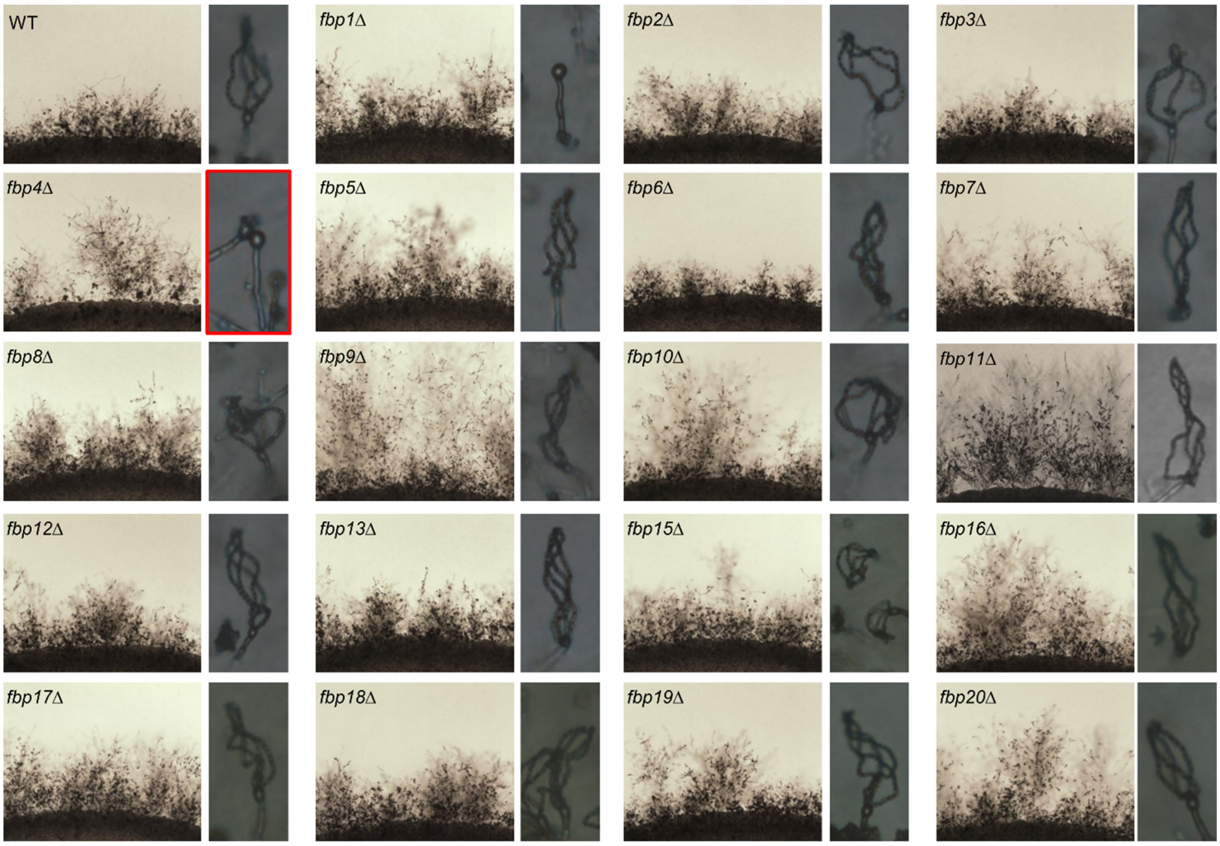

Figure S5. Mating filament production and sporulation of the wild type and mutants of F-box protein encoding genes in *C. neoformans*. Bilateral were performed on MS medium. Mating structures at ×40 magnification (left) and spores at ×200 magnification (right) were photographed after 2 weeks of incubation in the dark at 25°C.

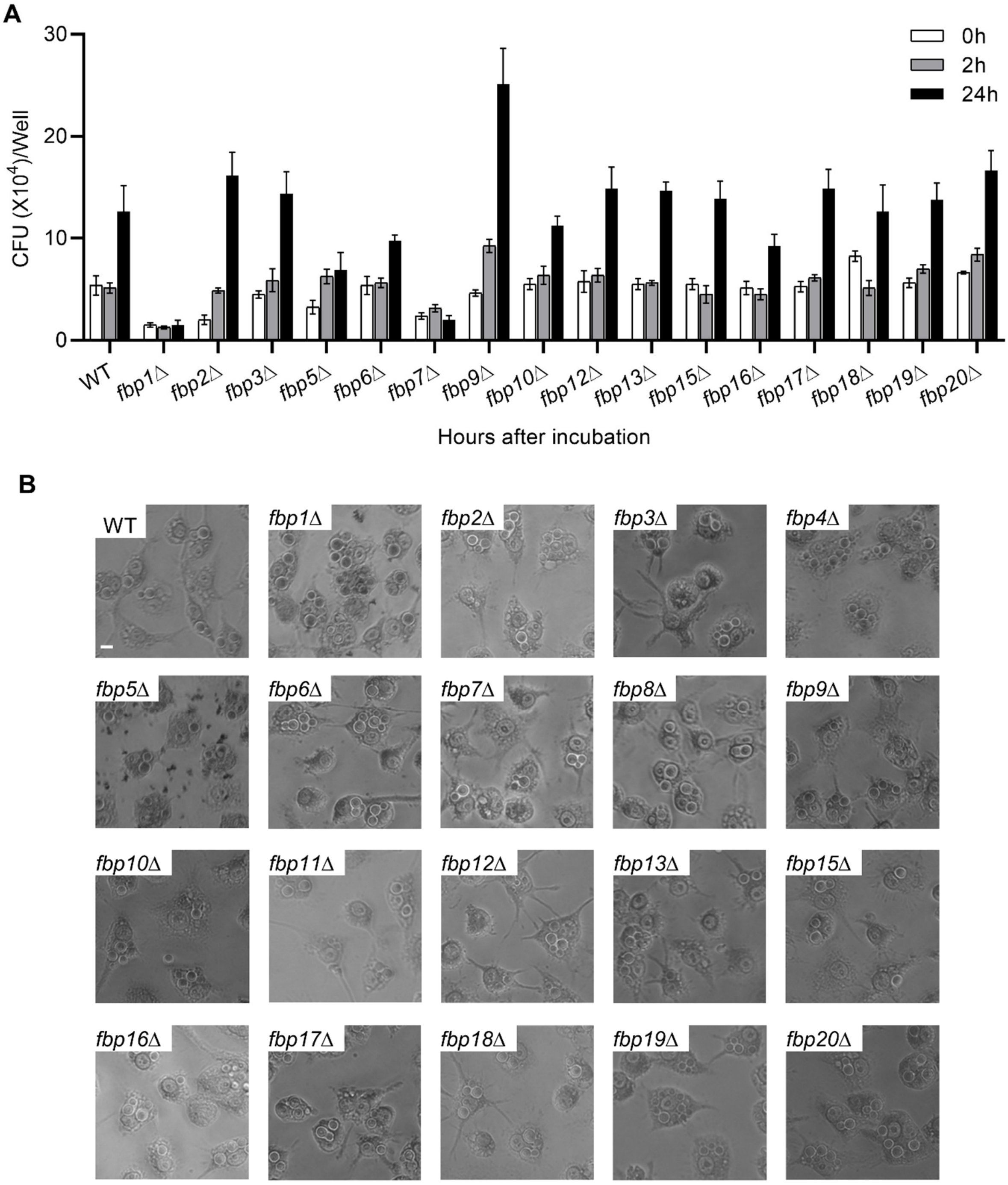

Figure S6. Killing assay and phagocytosis of the wild type and mutants of F-box protein encoding genes in *C. neoformans*. (A) Phagocytosis was allowed to occur for 2 h to measure both fungicidal and fungistatic activity. After each time interval, macrophages were lysed by adding sterile ddH_2O. Cells were counted and plated on YPD agar plates to determine CFU. (B) Typical field views of phagocytosis for each sample.
