# Supplementary material for: Multiple F-Box Proteins Collectively Regulate Cell Development and Pathogenesis in the Human Pathogen Cryptococcus neoformans
Source: J Fungi (Basel). 2022 Nov 29;8(12):1259. doi: 10.3390/jof8121259 (PMC9781138; doi:10.3390/jof8121259)
Supplement: Supplementary file 1 [file jof-08-01259-s001.zip › Table S1 Strains used in this study.pdf]

Table S1 Strains and plasmids used in this study

Strains	Genotype	Source/reference
H99	MATalpha	[1]
KN99alpha	MATalpha	[2]
KN99**a**	MAT**a**	[2]
CUX2	MATalpha *fbp1::NEO*	[3]
CUX3	MAT**a** *fbp1::NEO*	[3]
CUX403	MATalpha *fbp3::NAT*	[4]
CUX404	MATalpha *fbp11::NAT*	[4]
CUX405	MATalpha *fbp20::NAT*	[4]
CUX407	MATalpha *fbp5::NAT*	[4]
CUX776	MATalpha *fbp8::NAT*	[4]
CUX777	MATalpha *fbp10:NAT*	[4]
CUX778	MATalpha *fbp9::NAT*	[4]
CUX780	MATalpha *fbp4::NAT*	[4]
CUX781	MATalpha *fbp15::NAT*	[4]
CUX782	MATalpha *fbp16::NAT*	[4]
CUX783	MATalpha *fbp18NAT*	[4]
CUX1281	MATalpha *fbp17::NAT*	[4]
CUX1282	MATalpha *fbp2::NAT*	[4]
CUX1228	MAT**a** *fbp5::NAT*	This study
CUX1229	MAT**a** *fbp8::NAT*	This study
CUX1230	MAT**a** *fbp10::NAT*	This study
CUX1231	MAT**a** *fbp9::NAT*	This study
CUX1232	MAT**a** *fbp4::NAT*	This study
CUX1233	MAT**a** *fbp16::NAT*	This study
CUX1234	MAT**a** *fbp18::NAT*	This study
CUX1276	MAT**a** *fbp3::NAT*	This study
CUX1277	MAT**a** *fbp11::NAT*	This study
CUX1278	MAT**a** *fbp20::NAT*	This study
CUX1279	MAT**a** *fbp15::NAT*	This study
CUX1283	MATalpha *fbp19::NAT*	This study
CUX1284	MATalpha *fbp7::NAT*	This study
CUX1285	MATalpha *fbp12::NAT*	This study
CUX1286	MATalpha *fbp6::NAT*	This study
CUX1287	MATalpha *fbp13::NAT*	This study
CUX1297	MAT**a** *fbp2::NAT*	This study
CUX1298	MAT**a** *fbp6::NAT*	This study
CUX1299	MAT**a** *fbp7::NAT*	This study
CUX1300	MAT**a** *fbp12::NAT*	This study
CUX1301	MAT**a** *fbp13::NAT*	This study
CUX1302	MAT**a** *fbp17NAT*	This study
CUX1303	MAT**a** *fbp19NAT*	This study
CUX1319	MAT**a** *fbp4::NAT fbp11::NAT*	This study
CUX1320	MATalpha *fbp4::NAT fbp11::NAT*	This study
CUX1322	MATalpha *fbp11::NAT FBP11::NEO*	This study
CUX1323	MATalpha *fbp4::NAT FBP4::NEO*	This study
CUX1324	MATalpha *fbp8::NAT FBP8::NEO*	This study

Reference

1 Perfect, J. R., Schell, W. A. & Rinaldi, M. G. Uncommon invasive fungal pathogens in the acquired immunodeficiency syndrome. *J Med Vet Mycol* **31**, 175-179 (1993).

2 Nielsen, K. *et al.* Sexual cycle of Cryptococcus neoformans var. grubii and virulence of congenic a and alpha isolates. *Infect Immun* **71**, 4831-4841, doi:10.1128/iai.71.9.4831-4841.2003 (2003).

3 Liu, T. B. *et al.* The F-Box protein Fbp1 regulates sexual reproduction and virulence in *Cryptococcus neoformans*. *Eukaryot Cell* **10**, 791-802, doi:10.1128/EC.00004-11 (2011).

4 Chun, C. D. & Madhani, H. D. Applying genetics and molecular biology to the study of the human pathogen Cryptococcus neoformans. *Methods Enzymol* **470**, 797-831, doi:10.1016/S0076-6879(10)70033-1 (2010).
