# Supplementary material for: Multiple F-Box Proteins Collectively Regulate Cell Development and Pathogenesis in the Human Pathogen Cryptococcus neoformans
Source: J Fungi (Basel). 2022 Nov 29;8(12):1259. doi: 10.3390/jof8121259 (PMC9781138; doi:10.3390/jof8121259)
Supplement: Supplementary file 1 [file jof-08-01259-s001.zip › Table S2 Primers used in this study.pdf]

Table S2. Primers used in this study.

Primers	Sequences (5'-3')	Description
CX449	AACACCAGCACCAACACCAACA	*FBP11* F3
CX450a	TTGGCCTTGGCCTTTGTCTTGA	*FBP11* R3
CX1346	TTCTGATAACTCGCGAATCG	*FBP19* F1
CX1347	ctggccgtcgttttacAGATGAAGTTGCCATTGTCC	*FBP19* R1
CX1348	gtcatagctgtttcctgTCATCTTCCGAGCGTAATG	*FBP19* F2
CX1349	AGGGACATCTCGATGGATC	*FBP19* R2
CX1350	TCTCAGCAGATTCACCAGC	*FBP19* F3
CX1351	AGTGTAAGCTTGCGATATCC	*FBP19* R3
CX1352	ACAAGTGCACAGTCTTCTCC	*FBP19* F4
CX1353	ACAATGCCAAGATGACGAC	*FBP7* F1
CX1354	ctggccgtcgttttacAGGAGGAGTTATCTGGCAAG	*FBP7* R1
CX1355	gtcatagctgtttcctgACGAATTGTCACCAGAATG	*FBP7* F2
CX1356	ATCTCTTGTGCAGCCAACTC	*FBP7* R2
CX1357	TGGATCAATAGCTCAAGAACC	*FBP7* F3
CX1358	CACTGAAAGCATCTCCACAC	*FBP7* R3
CX1359	TGAATAACATCGTCCACGTC	*FBP7* F4
CX1360	ACTTTACTTCCCTCCTGCAG	*FBP12* F1/F3
CX1361	ctggccgtcgttttacACATACCTCCACCAGTCACC	*FBP12*/*FBP13* R1
CX1362	gtcatagctgtttcctgTGCAGACTAACGTTGAATGG	*FBP12* F2
CX1363	TCACATGCACCGTTTAGAAG	*FBP12* R2
CX1365	TCACAAACTTCGTCCCTGC	*FBP12* R4
CX1366	TGGTTTCAACTACGATCACC	*FBP12* F4
CX1367	ACTACGAGGAAAGAGTGGGTC	*FBP13* F1/F3
CX1368	TGTCAGGTTGGTCAAATTAGC	*FBP13* R4
CX1369	gtcatagctgtttcctgTCGAGTGACTTGTTTTGTGC	*FBP13* F2
CX1370	AATCCGCTAAACCGTGTATC	*FBP13* R2
CX1371	ATGGATGTGGAATGATTGTC	*FBP13*/*FBP12* R3
CX1373	TCCAAGCGAAGAGATACAATG	*FBP13* F4
CX1374	TGCCCGTGGTTATAAGACAG	*FBP14* F1
CX1375	ctggccgtcgttttacAGAAGGAGACATGGTGGGAG	*FBP13* R1
CX1376	gtcatagctgtttcctgTTGATATCACTCCATCCACG	*FBP13* F2
CX1377	AACTCGTCCTTTTCCTGTCC	*FBP13* R2
CX1378	ACAGTGAGCTCGATTGGTG	*FBP13* F3
CX1379	TCCATAACCCTCAAACACTC	*FBP13* R3
CX1380	AGGAACGTGTGTCATCTAGTC	*FBP13* F4
CX1381	TCCATGGCAGTAGTGATCC	*FBP6* F1
CX1382	ctggccgtcgttttacTCTGAACTGGAAGACAAAATG	*FBP6* R1
CX1383	gtcatagctgtttcctgTCGTCTGAGTGCATATTCTGG	*FBP6* F2
CX1384	TCACCTGATGAGCCTTGAG	*FBP6* R2

CX1385	TGAGGAGTTTGGCAATATCAG	*FBP6* F3
CX1386	TGGTTGTAACGTGTGTAGCTG	*FBP6* R3
CX1387	TGACTCACTGTTCGTCCTACC	*FBP6* F4
CX1925	TCCGAGCTCATCATTCTTAC	*ERG11* qRT-PCR F
CX1926	TGTTCGTGGTCGCTTTCAC	*ERG11* qRT-PCR R
CX1991	TCCATCAGCAAATCCACAAG	*FBP3* F3
CX1992	TCTTTCATCGTCTGCCCTG	*FBP3* R3
CX1993	TGGAGTGGATTCGGTACAG	*FBP4* F3
CX1994	TGGTCCCTTTCACAATGTC	*FBP4* R3
CX1995	TCCTATCCTTGCTCATTTGG	*FBP9* F3
CX1996	TTCCACGACTTGTCTGTCTC	*FBP9* R3
CX1997	AGGACAGGCTGTTGAAACG	*FBP10* F3
CX1998	TATTGAACAATTCCCCATCG	*FBP10* R3
CX1999	TCCCTATTATCAGCTCATCG	*FBP16* F3
CX2000	ATCCCATCTCTTACCCTCAG	*FBP16* R3
CX2001	AACTTCACCTGCCCATTATG	*FBP18* F3
CX2002	TAAGCATCTCACCCCGATTG	*FBP18* R3
CX2003	TCTATCCCAAGCTACCATGC	*FBP5* F3
CX2004	AATTCCTGAACGGCAATCG	*FBP5* R3
CX2005	ATACTATCAACTTTCGGTGG	*FBP15* F3
CX2006	ACCAATTCTCTCATGAAACC	*FBP15* R3
CX2007	TACCCACCTCAAGGATATGG	*FBP20* F3
CX2008	TCGTTCATAGGTATCTTTCG	*FBP20* R3
CX2009	AGGACGAAGAAGGCATGAAG	*FBP8* F3
CX2010	ACATTGCGCTTAACTTGGTC	*FBP8* R3
CX2061	taccgagctcggatccTCCTGATGTCCAAACGATTG	*FBP4* complement F
CX2062	cgttactagtggatccTATGCCGTCCAGTTACACTC	*FBP4* complement R
CX2063	taccgagctcggatccTCTAGCAAGAACCACAGTCC	*FBP11* complement F
CX2064	cgttactagtggatccTCTATCTGCTTATCTCTGCC	*FBP11* complement R
CX2065	taccgagctcggatccTCAGTCCACACCAAACAGC	*FBP8* complement F
CX2066	cgttactagtggatccAGCAAAGATTGACGATAATG	*FBP8* complement R
